# Supplementary figures and images for: Factors that affect migratory Western Atlantic red knots (Calidris canutus rufa) and their prey during spring staging on Virginia’s barrier islands
Source: PLoS One. 2022 Jul 1;17(7):e0270224. doi: 10.1371/journal.pone.0270224 (PMC9249208; doi:10.1371/journal.pone.0270224)

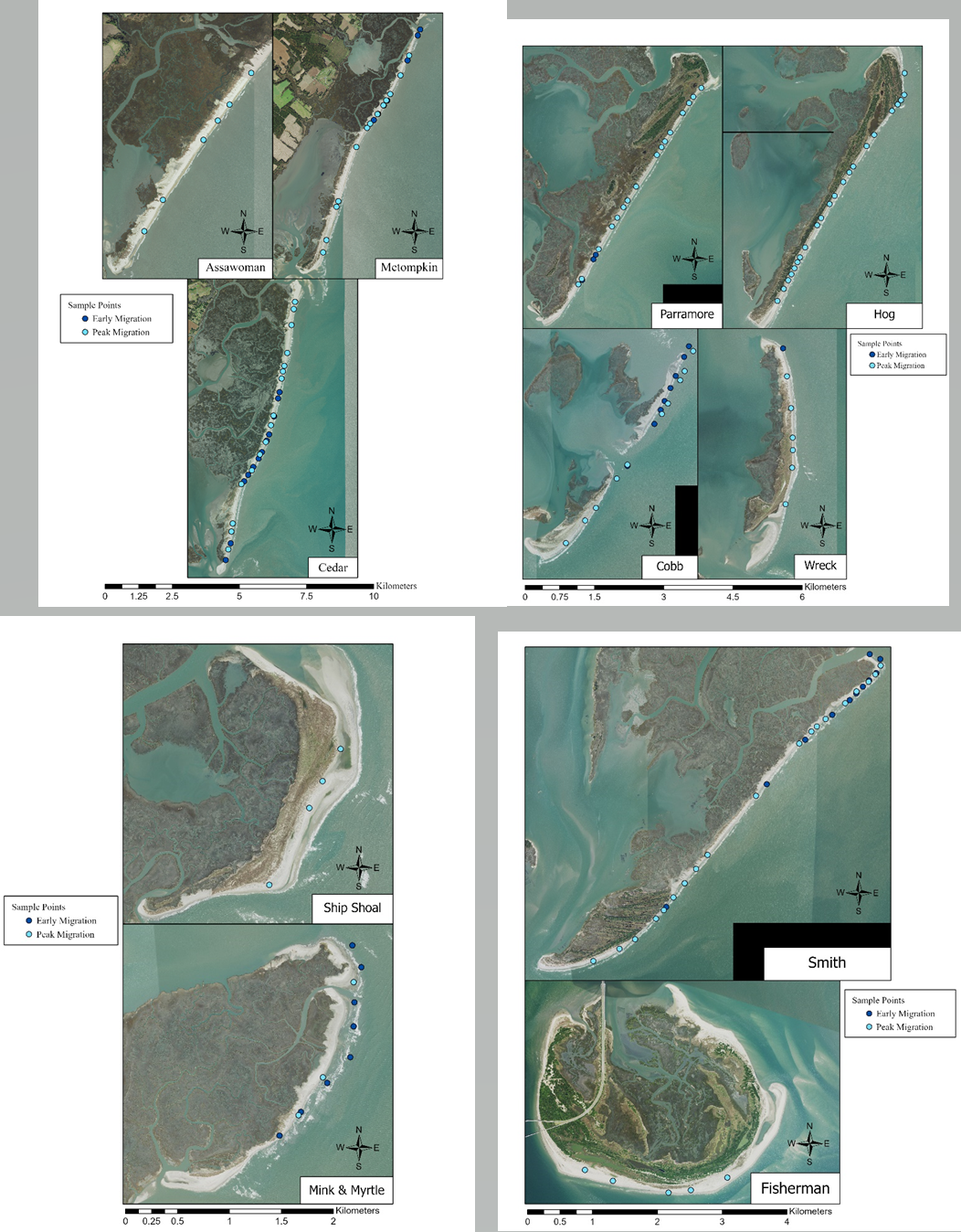

Supplement: S1 Fig — We sampled red knots and invertebrate prey at randomly generated points, each separated by at least 200m, on 11 barrier islands. Sample points collected during the early migration period (peat substrate only, May 14–20, 2018) are shown in dark blue whereas sample points during the peak migration period (peat and sand substrate, May 21–27, 2018) are shown in light blue on the images. From north to south, samples were collected on Assawoman Island, Metompkin Island, Cedar Island, Parramore Island, Hog Island, Cobb Island, Wreck Island, Ship Shoal Island, Mink and Myrtle Island, Smith Island and Fisherman Island. Basemap aerial island imagery from 2021 was taken from the United States Department of Agriculture’s National Agriculture Imagery Program (NAIP), available at [50] from the Aerial Photography Field Office. The geographic coordinates and details on all sample points from 2007–2018 can be found in [51]. (TIF) [file pone.0270224.s001.tif]
