# Supplementary material for: Factors that affect migratory Western Atlantic red knots (Calidris canutus rufa) and their prey during spring staging on Virginia’s barrier islands
Source: PLoS One. 2022 Jul 1;17(7):e0270224. doi: 10.1371/journal.pone.0270224 (PMC9249208; doi:10.1371/journal.pone.0270224)
Supplement: S1 Table — (DOCX) [file pone.0270224.s002.docx]

**S1 Table.** Mean red knot (*Calidris canutus rufa*) flock size per 100 m radius semicircle on the water line and mean abundance (organisms/m^2^ ± SE) of coquina clams (*Donax variabilis*), blue mussels (*Mytilus edulis*), crustaceans, miscellaneous prey, and all prey captured in 10 cm diameter x 3.5 cm deep cores on peat banks only early in red knot migration (May 14 – 20, 2008 – 2018; *n =* 457; ‘early’) and on sand and peat banks at the approximate peak of red knot migration (May 21 – 27, 2007 – 2018; *n =* 1,322; ‘peak’), Virginia’s barrier islands.

| **Year** | **Period** | ***n*** | ***Calidris canutus rufa*** | | ***Donax variabilis*** | | ***Mytilus edulis*** | | **Crustacean** | | **Misc. Prey^b^** | |
| --- | --- | --- | --- | --- | --- | --- | --- | --- | --- | --- | --- | --- |
|  |  |  | **x̅ ()^a^** | **SE** | **x̅ ()^a^** | **SE** | **x̅ ()^a^** | **SE** | **x̅ ()^a^** | **SE** | **x̅ ()^a^** | **SE** |
| 2007 | Early | . | . | . | . | . | . | . | . | . | . | . |
|  | Peak | 96 | 9.26(71) | 2.85 | 1484.87(36) | 291.68 | 49.09(93) | 45.17 | 2052.81(20) | 426.70 | 1963.90(45) | 760.46 |
| 2008 | Early | 61 | 5.05(42) | 1.67 | 14.62(56) | 6.72 | 3180.54*(39) | 1199.3 | 474.05(44) | 344.95 | 457.35*(32) | 119.17 |
|  | Peak | 98 | 12.03(72) | 3.67 | 617.44*(50) | 206.99 | 0(98) | 0.00 | 1243.99*(28) | 386.41 | 214.48(67) | 62.43 |
| 2009 | Early | 42 | 3.79(28) | 1.34 | 54.60(33) | 28.18 | 23190.78*(15) | 7437.8 | 5119.81(13) | 1466.47 | 3117.99*(6) | 645.30 |
|  | Peak | 99 | 14.14(69) | 4.91 | 599.63*(50) | 154.95 | 0.00(99) | 0.00 | 2618.54(22) | 1417.11 | 75.92(71) | 14.62 |
| 2010 | Early | . | . | . | . | . | . | . | . | . | . | . |
|  | Peak | 95 | 19.36(66) | 6.21 | 1745.89(26) | 396.13 | 710.69(90) | 703.93 | 1993.97(18) | 727.73 | 332.55(53) | 72.60 |
| 2011 | Early | 50 | 2.78(29) | 0.92 | 66.24(40) | 25.80 | 19602.55*(13) | 6701.6 | 56392.36*(7) | 12138.42 | 2845.9*(12) | 544.03 |
|  | Peak | 93 | 22.00(68) | 6.76 | 1925.90*(26) | 445.50 | 0.00(93) | 0.00 | 1865.63(18) | 372.13 | 276.69(68) | 136.23 |
| **Year** | **Period** | ***n*** | ***Calidris canutus rufa*** | | ***Donax variabilis*** | | ***Mytilus edulis*** | | **Crustacean** | | **Misc. Prey^b^** | |
|  |  |  | **x̅ ()^a^** | **SE** | **x̅ ()^a^** | **SE** | **x̅ ()^a^** | **SE** | **x̅ ()^a^** | **SE** | **x̅ ()^a^** | **SE** |
| 2012 | Early | 42 | 0.14(37) | 0.06 | 300.27(27) | 101.15 | 2826.81*(17) | 1084.6 | 4916.59(11) | 1396.40 | 3739.76(8) | 886.67 |
|  | Peak | 125 | 28.56*(89) | 11.4 | 1881.27*(53) | 806.93 | 9.17(124) | 9.17 | 2825.99(20) | 605.35 | 11080*(11) | 1663.6 |
| 2013 | Early | 43 | 18.10*(19) | 4.71 | 239.96(35) | 212.90 | 41424.97*(5) | 7723.5 | 19321.58*(6) | 6186.96 | 2956.60*(6) | 492.31 |
|  | Peak | 127 | 8.33(110) | 3.86 | 1569.79*(66) | 638.42 | 7156.82(110) | 2586.8 | 3388.33(29) | 736.41 | 2527.71(56) | 721.13 |
| 2014 | Early | 50 | 6.44*(30) | 2.41 | 40.76(46) | 26.85 | 20043.31*(5) | 4470.9 | 36636.94*(5) | 14192.64 | 4400.00*(6) | 727.22 |
|  | Peak | 129 | 9.60(113) | 5.02 | 779.14*(65) | 158.87 | 69.13(123) | 49.19 | 3210.40(24) | 663.31 | 223.18(92) | 75.15 |
| 2015 | Early | 46 | 10.57(31) | 4.26 | 138.47(33) | 45.12 | 10185.54*(6) | 2905.0 | 6380.50(10) | 3466.07 | 2254.22*(4) | 369.29 |
|  | Peak | 128 | 25.55(99) | 7.72 | 2875.20*(36) | 491.16 | 17.91(126) | 12.93 | 1391.32(22) | 151.15 | 19.90(117) | 6.99 |
| 2016 | Early | 39 | 7.67(30) | 3.06 | 101.26(27) | 32.14 | 3805.32*(2) | 602.51 | 5075.94*(2) | 1481.2 | 1538.46*(2) | 236.61 |
|  | Peak | 112 | 21.57(97) | 10.9 | 757.51*(48) | 136.76 | 18.19(109) | 13.31 | 3271.16(27) | 709.08 | 37.53(103) | 16.69 |
| 2017 | Early | 40 | 15.98*(18) | 4.44 | 73.25(34) | 41.28 | 4700.64*(11) | 1761.4 | 13257.96*(3) | 4007.12 | 2226.11*(5) | 417.55 |
|  | Peak | 109 | 14.25(78) | 5.92 | 1220.13*(44) | 256.83 | 72.45(103) | 46.68 | 3229.12(17) | 443.81 | 66.61(86) | 23.40 |
| 2018 | Early | 44 | 2.95(31) | 1.15 | 228.72(28) | 90.23 | 3679.79*(11) | 1013.4 | 20309.79*(3) | 8050.05 | 891.72*(13) | 182.24 |
|  | Peak | 111 | 16.77(86) | 8.36 | 907.79*(55) | 169.10 | 224.94(98) | 79.79 | 3874.45(17) | 890.52 | 182.48(88) | 53.45 |
| TOTAL | Early | 457 | 7.15(295) | 0.90 | 119.86(359) | 25.18 | 13166*^†^(124) | 1482.9 | 17173*^†^(104) | 2443.73 | 2401*^†^(94) | 169.16 |
|  | Peak | 1,322 | 16.88*(1,018)^†^ | 2.09 | 1383.54*^†^(555) | 125.32 | 777.91(1,266) | 259.31 | 2621.29(262) | 199.65 | 1545.7(857) | 200.61 |

^a^ x̅ () = Number in parentheses following each mean value represents the number of 0 counts for each category during its defined period and year.

^b^ Misc. Prey = Miscellaneous Prey = Sum of horseshoe crab eggs (*Limulus polyphemus*), angel wing clams (*Cyrtopleura costata*), and other organisms (e.g., insect larvae, snails, worms).

^*^ = Difference (p < 0.05) between means of early migration period vs. peak migration period based on Wilcoxon rank sum tests between peat and peak migration period within a given year. ^*^placed with the larger mean.

^†^ = Difference (p < 0.05) between mean red knot or prey abundances based on Wilcoxon rank sum tests during peat bank period and peak migration period across all years. ^†^ placed with the larger mean.
