# Supplementary material for: Factors that affect migratory Western Atlantic red knots (Calidris canutus rufa) and their prey during spring staging on Virginia’s barrier islands
Source: PLoS One. 2022 Jul 1;17(7):e0270224. doi: 10.1371/journal.pone.0270224 (PMC9249208; doi:10.1371/journal.pone.0270224)
Supplement: S2 Table — (DOCX) [file pone.0270224.s003.docx]

**S2 Table.** Mean red knot (*Calidris canutus rufa*) flock size and standard errors per 100 m radius semicircle on the water line on peat banks early in red knot migration (May 14 – 20, 2008 – 2018; *n =* 457; ‘early’) and on sand and peat banks at the approximate peak of red knot migration (May 21 – 27, 2007 – 2018; *n =* 1,322; ‘peak’), Virginia’s barrier islands.

| **Period** | **Year** | **Sample Size** | **Mean Red Knot Flock Size** | **Standard Deviation** | **Standard Error** | **Variance** |
| --- | --- | --- | --- | --- | --- | --- |
| Early | 2008 | 61 | 5.05 | 13.08 | 0.65 | 171.11 |
|  | 2009 | 42 | 3.79 | 8.69 | 0.58 | 75.44 |
|  | 2011 | 50 | 2.78 | 6.53 | 0.39 | 42.71 |
|  | 2012 | 42 | 0.14 | 0.42 | 0.02 | 0.17 |
|  | 2013 | 43 | 18.16 | 30.90 | 2.77 | 954.95 |
|  | 2014 | 50 | 6.44 | 17.43 | 0.91 | 303.92 |
|  | 2015 | 46 | 10.57 | 28.90 | 1.56 | 835.32 |
|  | 2016 | 39 | 7.67 | 19.14 | 1.23 | 366.33 |
|  | 2017 | 40 | 15.98 | 28.07 | 2.53 | 787.87 |
|  | 2018 | 44 | 2.95 | 7.62 | 0.45 | 58.00 |
| Peak | 2007 | 96 | 9.26 | 27.90 | 0.95 | 778.45 |
|  | 2008 | 98 | 12.03 | 36.37 | 1.22 | 1,322.79 |
|  | 2009 | 99 | 14.14 | 48.84 | 1.42 | 2,384.94 |
|  | 2010 | 95 | 19.36 | 60.50 | 1.99 | 3,660.30 |
|  | 2011 | 93 | 22.00 | 65.14 | 2.28 | 4,243.72 |
|  | 2012 | 125 | 28.56 | 127.47 | 2.55 | 16,247.46 |
|  | 2013 | 127 | 8.33 | 43.54 | 0.74 | 1,895.41 |
|  | 2014 | 129 | 9.60 | 56.96 | 0.85 | 3,244.44 |
|  | 2015 | 128 | 25.55 | 87.30 | 2.26 | 7,621.78 |
|  | 2016 | 112 | 21.57 | 115.76 | 2.04 | 13,401.47 |
|  | 2017 | 109 | 14.25 | 61.83 | 1.36 | 3,822.63 |
|  | 2018 | 111 | 16.77 | 88.09 | 1.59 | 7,760.11 |
