# Supplementary material for: Factors that affect migratory Western Atlantic red knots (Calidris canutus rufa) and their prey during spring staging on Virginia’s barrier islands
Source: PLoS One. 2022 Jul 1;17(7):e0270224. doi: 10.1371/journal.pone.0270224 (PMC9249208; doi:10.1371/journal.pone.0270224)
Supplement: S3 Table — Highly correlated covariate combinations are represented by values > 0.7 and < -0.7 (Booth et al. 1994, Anderson et al. 2001). (DOCX) [file pone.0270224.s004.docx]

**S3 Table.** Pearson correlation coefficients for all continuous covariates on peat banks early in red knot migration (May 14 – 20, 2008 – 2018; *n =* 457; ‘early’) and on sand and peat banks at the approximate peak of red knot migration (May 21 – 27, 2007 – 2018; *n =* 1,322; ‘peak’), Virginia’s barrier islands. Highly correlated covariate combinations are represented by values > 0.7 and < -0.7 [70-71].

| **Period** | **Covariate** | **Red Knot^a^** | **Distance to Roost^b^** | **TDF Count^c^** | **Blue Mussel^d^** | **Crustacean^d^** | **Coquina Clam^d^** | **Miscellaneous Prey^d^** | **All Prey^d^** |
| --- | --- | --- | --- | --- | --- | --- | --- | --- | --- |
| Early | Red Knot | 1.00 | 0.17 | -0.07 | 0.23 | 0.17 | 0.04 | 0.21 | 0.25 |
|  | Distance to Roost | 0.17 | 1.00 | -0.02 | 0.37 | 0.13 | 0.01 | 0.35 | 0.30 |
|  | TDF Count | -0.07 | -0.02 | 1.00 | -0.05 | -0.17 | -0.07 | 0.06 | -0.15 |
|  | Blue Mussel | 0.23 | 0.37 | -0.05 | 1.00 | 0.17 | -0.03 | 0.50 | 0.63 |
|  | Crustacean | 0.17 | 0.13 | -0.17 | 0.17 | 1.00 | 0.12 | 0.13 | 0.87 |
|  | Coquina Clam | 0.04 | 0.01 | -0.07 | -0.03 | 0.12 | 1.00 | -0.01 | 0.09 |
|  | Miscellaneous Prey | 0.21 | 0.35 | 0.06 | 0.50 | 0.13 | -0.01 | 1.00 | 0.39 |
|  | All Prey | 0.25 | 0.30 | -0.15 | 0.63 | 0.87 | 0.09 | 0.39 | 1.00 |

| Peak | Red Knot | 1.00 | -0.13 | -0.01 | -0.02 | 0.01 | 0.11 | 0.10 | 0.07 |
| --- | --- | --- | --- | --- | --- | --- | --- | --- | --- |
|  | Distance to Roost | -0.13 | 1.00 | -0.03 | 0.10 | -0.07 | -0.06 | -0.05 | -0.01 |
|  | TDF Count | -0.01 | -0.03 | 1.00 | -0.09 | -0.05 | -0.02 | 0.04 | -0.06 |
|  | Blue Mussel | -0.02 | 0.10 | -0.09 | 1.00 | 0.21 | -0.02 | 0.02 | 0.68 |
|  | Crustacean | 0.01 | -0.07 | -0.05 | 0.21 | 1.00 | 0.02 | 0.08 | 0.62 |
|  | Coquina Clam | 0.11 | -0.06 | -0.02 | -0.02 | 0.02 | 1.00 | 0.03 | 0.29 |
|  | Miscellaneous Prey | 0.10 | -0.05 | 0.04 | 0.02 | 0.08 | 0.03 | 1.00 | 0.51 |
|  | All Prey | 0.07 | -0.01 | -0.06 | 0.68 | 0.62 | 0.29 | 0.51 | 1.00 |

^a^ Red Knot = At randomly generated sampling points on Virginia’s barrier islands, we counted the number of non-flying red knots within a 100 m semicircle radius of each point.

^b^ Distance to Roost = We calculated distance to roost as the distance between sampling points and locations presumed to be used for night roosting by red knots on Chimney Pole and Wreck Island.

^c^ Tierra del Fuego Count = Counts of red knots using Tierra del Fuego wintering grounds by year (i.e., as an index for the total number of red knots in the flyway.

^d^ Blue Mussel, Crustacean, Coquina Clam, Miscellaneous Prey, All Prey = At randomly generated sampling points on Virginia’s barrier islands, we sampled prey availability by collecting a core sample of the substrate at the water-line at each sampling point using a section of PVC piping (10 cm diameter x 3.5 cm deep). The cores’ depth represented the approximate length of a red knot’s bill, so that we sampled only prey that red knots would be able to access while foraging. Miscellaneous Prey = the sum of horseshoe crab eggs (*Limulus polyphemus*), angel wing clams (*Cyrtopleura costata*), and other organisms (e.g., insect larvae, snails, worms). All Prey = the sum of coquina clams + blue mussels + crustaceans + miscellaneous prey.
