# Supplementary material for: Factors that affect migratory Western Atlantic red knots (Calidris canutus rufa) and their prey during spring staging on Virginia’s barrier islands
Source: PLoS One. 2022 Jul 1;17(7):e0270224. doi: 10.1371/journal.pone.0270224 (PMC9249208; doi:10.1371/journal.pone.0270224)
Supplement: S4 Table — All models had the same covariates on both the zero-inflated and count processes and contained “Island” and “Year” as random effects. (DOCX) [file pone.0270224.s005.docx]

**S4 Table.** Full model sets for zero-inflated negative binomial mixed-effects regression models predicting red knot presence and flock size on peat banks early in red knot migration (May 14 – 20, 2008 – 2018, except 2010; *n* = 457; ‘early’), and on sand and peat substrates at the approximate peak of red knot migration (May 21 – 27, 2007 – 2018; *n* = 1,322; ‘peak’), Virginia’s barrier islands. All models had the same covariates on both the zero-inflated and count processes and contained “Island” and “Year” as random effects.

| **Period** | **Model** | **DF^a^** | **AIC*_c_*^b^** | **ΔAIC*_c_*^c^** | ***w_i_*^d^** | **LL^e^** | **GOF^e^** |
| --- | --- | --- | --- | --- | --- | --- | --- |
| Early | Coquina Clam + Crustacean + Miscellaneous Prey^g^ + Tide | 17 | 1,743.25 | 0.00 | 0.30 | -853.93 | 0.08 |
|  | Crustacean + Miscellaneous Prey^g^ | 11 | 1,744.50 | 1.25 | 0.16 | -860.96 | 0.07 |
|  | All Prey^h^ + Tide | 13 | 1,745.28 | 2.03 | 0.11 | -859.23 | 0.07 |
|  | Blue Mussel + Coquina Clam + Crustacean + Miscellaneous Prey^g^ + Tide | 19 | 1,745.46 | 2.20 | 0.10 | -852.86 | 0.08 |
|  | Blue Mussel + Crustacean + Miscellaneous Prey^g^ | 13 | 1,745.93 | 2.68 | 0.08 | -859.55 | 0.07 |
|  | Crustacean + Miscellaneous Prey^g^ + Distance to Roost | 13 | 1,746.00 | 2.75 | 0.08 | -859.59 | 0.07 |
|  | All Prey^h^ + Tide + Distance to Roost | 15 | 1,746.68 | 3.42 | 0.05 | -857.79 | 0.07 |
|  | Coquina Clam + Crustacean + Miscellaneous Prey^g^ | 13 | 1,748.45 | 5.20 | 0.02 | -860.82 | 0.07 |
|  | Miscellaneous Prey^g^ + Tide | 13 | 1,748.81 | 5.56 | 0.02 | -860.99 | 0.07 |
|  | All Prey^h^ + Tide + TDF Count^i^ | 15 | 1,749.02 | 5.76 | 0.02 | -858.96 | 0.07 |
|  | Blue Mussel + Coquina Clam + Crustacean + Miscellaneous Prey^g^ | 15 | 1,749.89 | 6.63 | 0.01 | -859.40 | 0.07 |
|  | Crustacean + Miscellaneous Prey^g^ + Distance to Roost + TDF Count^i^ | 15 | 1,749.92 | 6.67 | 0.01 | -859.42 | 0.07 |
|  | Blue Mussel + Miscellaneous Prey^g^ + Tide | 15 | 1,749.94 | 6.68 | 0.01 | -859.42 | 0.07 |
|  | Coquina Clam + Crustacean + Miscellaneous Prey^g^ + Distance to Roost | 15 | 1,749.96 | 6.70 | 0.01 | -859.43 | 0.07 |
|  | All Prey^h^ + Distance to Roost + TDF Count^i^ + Tide | 17 | 1,750.41 | 7.16 | 0.01 | -857.51 | 0.07 |
|  | All Prey^h^ | 9 | 1,751.01 | 7.75 | 0.01 | -866.30 | 0.06 |
|  | All Prey^h^ + Distance to Roost | 11 | 1,751.45 | 8.19 | 0.00 | -864.43 | 0.06 |
|  | Coquina Clam + Miscellaneous Prey^g^ + Tide | 15 | 1,752.34 | 9.09 | 0.00 | -860.63 | 0.07 |
|  | Coquina Clam + Crustacean + Miscellaneous Prey^g^ + TDF Count^i^ | 15 | 1,752.41 | 9.16 | 0.00 | -860.66 | 0.07 |
|  | Blue Mussel + Crustacean + Tide | 15 | 1,753.80 | 10.55 | 0.00 | -861.36 | 0.07 |
|  | Blue Mussel + Coquina Clam + Crustacean + Miscellaneous Prey^g^ + TDF Count^i^ | 17 | 1,753.84 | 10.59 | 0.00 | -859.22 | 0.07 |
|  | All Prey^h^ + TDF Count^i^ | 11 | 1,754.88 | 11.63 | 0.00 | -866.14 | 0.06 |
|  | Blue Mussel + Coquina Clam + Crustacean + Tide | 17 | 1,757.20 | 13.94 | 0.00 | -860.90 | 0.07 |
|  | Crustacean + Tide | 13 | 1,757.29 | 14.03 | 0.00 | -865.23 | 0.06 |
|  | Blue Mussel + Crustacean | 11 | 1,759.82 | 16.57 | 0.00 | -868.61 | 0.06 |
|  | Blue Mussel + Crustacean + Distance to Roost | 13 | 1,760.36 | 17.10 | 0.00 | -866.77 | 0.06 |
|  | Miscellaneous Prey^g^ | 9 | 1,760.37 | 17.11 | 0.00 | -870.98 | 0.06 |
|  | Blue Mussel + Miscellaneous Prey^g^ | 11 | 1,760.67 | 17.42 | 0.00 | -869.04 | 0.06 |
|  | Coquina Clam + Crustacean + Tide | 15 | 1,760.90 | 17.64 | 0.00 | -864.90 | 0.06 |
|  | Blue Mussel + Miscellaneous Prey^g^ + Distance to Roost | 13 | 1,761.36 | 18.11 | 0.00 | -867.27 | 0.06 |
|  | Miscellaneous Prey^g^ + Distance to Roost | 11 | 1,762.35 | 19.10 | 0.00 | -869.88 | 0.06 |
|  | Blue Mussel + Coquina Clam + Crustacean | 13 | 1,763.05 | 19.80 | 0.00 | -868.11 | 0.06 |
|  | Blue Mussel + Coquina Clam + Miscellaneous Prey^g^ | 13 | 1,763.34 | 20.09 | 0.00 | -868.26 | 0.06 |
|  | Coquina Clam + Miscellaneous Prey^g^ | 11 | 1,763.42 | 20.17 | 0.00 | -870.41 | 0.06 |
|  | Miscellaneous Prey^g^ + TDF Count^i^ | 11 | 1,763.69 | 20.44 | 0.00 | -870.55 | 0.06 |
|  | Blue Mussel + Crustacean + TDF Count^i^ | 13 | 1,763.77 | 20.52 | 0.00 | -868.48 | 0.06 |
|  | Blue Mussel + Coquina Clam + Crustacean + Distance to Roost | 15 | 1,763.88 | 20.63 | 0.00 | -866.40 | 0.06 |
|  | Blue Mussel + Miscellaneous Prey^g^ + TDF Count^i^ | 13 | 1,764.30 | 21.05 | 0.00 | -868.74 | 0.06 |
|  | Coquina Clam + Miscellaneous Prey^g^ + Distance to Roost | 13 | 1,765.45 | 22.19 | 0.00 | -869.31 | 0.06 |
|  | Coquina Clam + Miscellaneous Prey^g^ + TDF Count^i^ | 13 | 1,766.90 | 23.64 | 0.00 | -870.04 | 0.06 |
|  | Blue Mussel + Coquina Clam + Crustacean + TDF Count^i^ | 15 | 1,766.98 | 23.73 | 0.00 | -867.95 | 0.06 |
|  | Crustacean | 9 | 1,770.50 | 27.25 | 0.00 | -876.05 | 0.05 |
|  | Blue Mussel + Tide | 13 | 1,770.93 | 27.67 | 0.00 | -872.05 | 0.06 |
|  | Blue Mussel + Distance to Roost + Tide | 15 | 1,771.96 | 28.71 | 0.00 | -870.44 | 0.06 |
|  | Crustacean + Distance to Roost | 11 | 1,772.79 | 29.54 | 0.00 | -875.10 | 0.05 |
|  | Coquina Clam + Crustacean | 11 | 1,773.46 | 30.21 | 0.00 | -875.43 | 0.05 |
|  | Blue Mussel + Coquina Clam + Tide | 15 | 1,773.72 | 30.46 | 0.00 | -871.31 | 0.06 |
|  | Crustacean + TDF Count^i^ | 11 | 1,774.30 | 31.05 | 0.00 | -875.85 | 0.05 |
|  | Coquina Clam + Crustacean + Distance to Roost | 13 | 1,775.83 | 32.57 | 0.00 | -874.50 | 0.05 |
|  | Coquina Clam + Crustacean + TDF Count^i^ | 13 | 1,777.10 | 33.85 | 0.00 | -875.14 | 0.05 |
|  | Tide | 11 | 1,781.17 | 37.92 | 0.00 | -879.29 | 0.05 |
|  | Coquina Clam + Tide | 13 | 1,784.47 | 41.22 | 0.00 | -878.82 | 0.05 |
|  | Blue Mussel + Distance to Roost | 11 | 1,785.03 | 41.78 | 0.00 | -881.22 | 0.05 |
|  | Blue Mussel | 9 | 1,785.04 | 41.78 | 0.00 | -883.32 | 0.04 |
|  | Blue Mussel + Coquina Clam | 11 | 1,786.66 | 43.40 | 0.00 | -882.03 | 0.05 |
|  | Blue Mussel + Coquina Clam + Distance to Roost | 13 | 1,786.79 | 43.53 | 0.00 | -879.98 | 0.05 |
|  | Blue Mussel + Distance to Roost + TDF Count^i^ | 13 | 1,788.76 | 45.51 | 0.00 | -880.97 | 0.05 |
|  | Blue Mussel + TDF Count^i^ | 11 | 1,788.83 | 45.57 | 0.00 | -883.12 | 0.04 |
|  | Blue Mussel + Coquina Clam + TDF Count^i^ | 13 | 1,790.60 | 47.34 | 0.00 | -881.89 | 0.05 |
|  | Coquina Clam | 9 | 1,809.39 | 66.13 | 0.00 | -895.49 | 0.03 |
|  | TDF Count^i^ | 9 | 1,809.83 | 66.57 | 0.00 | -895.71 | 0.03 |
|  | Distance to Roost | 9 | 1,810.66 | 67.40 | 0.00 | -896.13 | 0.03 |
|  | Coquina Clam + TDF Count^i^ | 11 | 1,812.85 | 69.60 | 0.00 | -895.13 | 0.03 |
|  | Coquina Clam + Distance to Roost | 11 | 1,813.56 | 70.31 | 0.00 | -895.48 | 0.03 |
|  | Null | 2 | 1,852.19 | 108.94 | 0.00 | -924.08 | 0.00 |
| Peak | Blue Mussel + Coquina Clam + Crustacean + TDF Count^i^ | 15 | 4,103.66 | 0.00 | 0.24 | -2,036.65 | 0.07 |
|  | Blue Mussel + Coquina Clam + Crustacean + Miscellaneous Prey^g^ + Distance to Roost | 25 | 4,104.14 | 0.48 | 0.19 | -2,026.57 | 0.08 |
|  | Blue Mussel + Coquina Clam + Crustacean + Miscellaneous Prey^g^ + TDF Count^i^ | 17 | 4,104.21 | 0.55 | 0.18 | -2,034.87 | 0.07 |
|  | Blue Mussel + Coquina Clam + TDF Count^i^ | 13 | 4,104.88 | 1.23 | 0.13 | -2,039.30 | 0.07 |
|  | Blue Mussel + Coquina Clam + Distance to Roost | 13 | 4,106.03 | 2.37 | 0.07 | -2,039.88 | 0.07 |
|  | Blue Mussel + Coquina Clam + Crustacean + Distance to Roost | 15 | 4,106.20 | 2.54 | 0.07 | -2,037.92 | 0.07 |
|  | Blue Mussel + Coquina Clam + Crustacean + Miscellaneous Prey^g^ + Distance to Roost | 17 | 4,106.70 | 3.05 | 0.05 | -2,036.12 | 0.07 |
|  | Blue Mussel + Coquina Clam + Crustacean | 13 | 4,109.37 | 5.71 | 0.01 | -2,041.54 | 0.07 |
|  | Blue Mussel + Coquina Clam | 11 | 4,109.50 | 5.84 | 0.01 | -2,043.65 | 0.07 |
|  | Blue Mussel + Coquina Clam + Miscellaneous Prey^g^ | 13 | 4,110.38 | 6.72 | 0.01 | -2,042.05 | 0.07 |
|  | Blue Mussel + Coquina Clam + Crustacean + Miscellaneous Prey^g^ | 15 | 4,110.39 | 6.73 | 0.01 | -2,040.01 | 0.07 |
|  | Blue Mussel + Coquina Clam + Crustacean + Tide | 19 | 4,111.02 | 7.36 | 0.01 | -2,036.22 | 0.07 |
|  | Blue Mussel + Coquina Clam + Crustacean + Miscellaneous Prey^g^ + Tide | 21 | 4,112.11 | 8.45 | 0.00 | -2,034.70 | 0.07 |
|  | Blue Mussel + Coquina Clam + Tide | 17 | 4,113.15 | 9.49 | 0.00 | -2,039.34 | 0.07 |
|  | Coquina Clam + Crustacean + TDF Count^i^ | 13 | 4,126.01 | 22.35 | 0.00 | -2,049.86 | 0.07 |
|  | Coquina Clam + Crustacean + Miscellaneous Prey^g^ + TDF Count^i^ | 15 | 4,126.70 | 23.04 | 0.00 | -2,048.17 | 0.07 |
|  | Coquina Clam + Crustacean + Distance to Roost | 13 | 4,127.27 | 23.61 | 0.00 | -2,050.50 | 0.07 |
|  | Coquina Clam + Crustacean + Miscellaneous Prey^g^ + Distance to Roost | 15 | 4,127.79 | 24.13 | 0.00 | -2,048.71 | 0.07 |
|  | Coquina Clam + TDF Count^i^ | 11 | 4,130.15 | 26.49 | 0.00 | -2,053.97 | 0.06 |
|  | Coquina Clam + Distance to Roost | 11 | 4,130.54 | 26.88 | 0.00 | -2,054.17 | 0.06 |
|  | Coquina Clam + Crustacean | 11 | 4,130.58 | 26.92 | 0.00 | -2,054.19 | 0.06 |
|  | Coquina Clam + Miscellaneous Prey^g^ + TDF Count^i^ | 13 | 4,130.73 | 27.07 | 0.00 | -2,052.22 | 0.06 |
|  | Coquina Clam + Miscellaneous Prey^g^ + Distance to Roost | 13 | 4,130.76 | 27.10 | 0.00 | -2,052.24 | 0.06 |
|  | Coquina Clam + Crustacean + Miscellaneous Prey^g^ | 13 | 4,131.62 | 27.96 | 0.00 | -2,052.67 | 0.06 |
|  | Coquina Clam + Crustacean + Tide | 17 | 4,132.16 | 28.50 | 0.00 | -2,048.84 | 0.07 |
|  | Coquina Clam + Crustacean + Miscellaneous Prey^g^ + Tide | 19 | 4,133.34 | 29.68 | 0.00 | -2,047.38 | 0.07 |
|  | Coquina Clam | 9 | 4,133.73 | 30.07 | 0.00 | -2,057.80 | 0.06 |
|  | Coquina Clam + Miscellaneous Prey^g^ | 11 | 4,134.52 | 30.86 | 0.00 | -2,056.16 | 0.06 |
|  | Coquina Clam + Tide | 15 | 4,137.13 | 33.47 | 0.00 | -2,053.38 | 0.06 |
|  | Coquina Clam + Miscellaneous Prey^g^ + Tide | 17 | 4,138.06 | 34.40 | 0.00 | -2,051.79 | 0.06 |
|  | All Prey^h^ + Distance to Roost + Tide + TDF Count^i^ | 19 | 4,161.36 | 57.70 | 0.00 | -2,061.39 | 0.06 |
|  | All Prey^h^ + Tide + Distance to Roost | 17 | 4,162.13 | 58.47 | 0.00 | -2,063.83 | 0.06 |
|  | All Prey^h^ + Tide + TDF Count^i^ | 17 | 4,164.24 | 60.58 | 0.00 | -2,064.88 | 0.06 |
|  | All Prey^h^ + Distance to Roost | 11 | 4,164.59 | 60.93 | 0.00 | -2,071.19 | 0.06 |
|  | All Prey^h^ + Tide | 15 | 4,164.73 | 61.08 | 0.00 | -2,067.18 | 0.06 |
|  | All Prey^h^ + TDF Count^i^ | 11 | 4,167.49 | 63.83 | 0.00 | -2,072.64 | 0.06 |
|  | All Prey^h^ | 9 | 4,168.76 | 65.10 | 0.00 | -2,075.31 | 0.05 |
|  | Blue Mussel + Distance to Roost + TDF Count^i^ | 13 | 4,177.36 | 73.70 | 0.00 | -2,075.54 | 0.05 |
|  | Blue Mussel + Crustacean + TDF Count^i^ | 13 | 4,178.73 | 75.07 | 0.00 | -2,076.23 | 0.05 |
|  | Blue Mussel + Miscellaneous Prey^g^ + Distance to Roost | 13 | 4,179.01 | 75.35 | 0.00 | -2,076.36 | 0.05 |
|  | Blue Mussel + Miscellaneous Prey^g^ + TDF Count^i^ | 13 | 4,179.60 | 75.94 | 0.00 | -2,076.66 | 0.05 |
|  | Blue Mussel + Crustacean + Distance to Roost | 13 | 4,179.64 | 75.99 | 0.00 | -2,076.68 | 0.05 |
|  | Blue Mussel + Distance to Roost | 11 | 4,180.02 | 76.37 | 0.00 | -2,078.91 | 0.05 |
|  | Blue Mussel + TDF Count^i^ | 11 | 4,180.42 | 76.76 | 0.00 | -2,079.11 | 0.05 |
|  | Blue Mussel + Crustacean + Tide | 17 | 4,180.73 | 77.08 | 0.00 | -2,073.13 | 0.05 |
|  | Blue Mussel + Crustacean + Miscellaneous Prey^g^ | 13 | 4,181.94 | 78.28 | 0.00 | -2,077.83 | 0.05 |
|  | Blue Mussel + Distance to Roost + Tide | 17 | 4,182.21 | 78.56 | 0.00 | -2,073.87 | 0.05 |
|  | Blue Mussel + Crustacean | 11 | 4,182.33 | 78.67 | 0.00 | -2,080.06 | 0.05 |
|  | Blue Mussel + Miscellaneous Prey^g^ | 11 | 4,182.58 | 78.92 | 0.00 | -2,080.19 | 0.05 |
|  | Blue Mussel | 9 | 4,182.90 | 79.24 | 0.00 | -2,082.38 | 0.05 |
|  | Blue Mussel + Tide | 15 | 4,183.75 | 80.10 | 0.00 | -2,076.69 | 0.05 |
|  | Blue Mussel + Miscellaneous Prey^g^ + Tide | 17 | 4,183.77 | 80.12 | 0.00 | -2,074.65 | 0.05 |
|  | Crustacean + Miscellaneous Prey^g^ + Distance to Roost + TDF Count^i^ | 15 | 4,192.57 | 88.91 | 0.00 | -2,081.10 | 0.05 |
|  | Crustacean + Miscellaneous Prey^g^ + Distance to Roost | 13 | 4,195.90 | 92.24 | 0.00 | -2,084.81 | 0.05 |
|  | Crustacean + Miscellaneous Prey^g^ + Distance to Roost + Tide | 19 | 4,196.29 | 92.64 | 0.00 | -2,078.86 | 0.05 |
|  | Crustacean + Distance to Roost | 11 | 4,196.94 | 93.28 | 0.00 | -2,087.37 | 0.05 |
|  | Crustacean + TDF Count^i^ | 11 | 4,197.72 | 94.06 | 0.00 | -2,087.76 | 0.05 |
|  | Crustacean + Tide | 15 | 4,198.78 | 95.12 | 0.00 | -2,084.21 | 0.05 |
|  | Miscellaneous Prey^g^ + Distance to Roost | 11 | 4,198.82 | 95.16 | 0.00 | -2,088.31 | 0.05 |
|  | Crustacean + Miscellaneous Prey^g^ | 11 | 4,199.74 | 96.09 | 0.00 | -2,088.77 | 0.05 |
|  | Distance to Roost | 9 | 4,200.01 | 96.35 | 0.00 | -2,090.94 | 0.05 |
|  | Crustacean | 9 | 4,200.19 | 96.54 | 0.00 | -2,091.03 | 0.05 |
|  | Miscellaneous Prey^g^ + TDF Count^i^ | 11 | 4,200.66 | 97.00 | 0.00 | -2,089.23 | 0.05 |
|  | TDF Count^i^ | 9 | 4,201.45 | 97.79 | 0.00 | -2,091.66 | 0.05 |
|  | Miscellaneous Prey^g^ | 9 | 4,202.38 | 98.72 | 0.00 | -2,092.12 | 0.05 |
|  | Miscellaneous Prey^g^ + Tide | 15 | 4,203.45 | 99.80 | 0.00 | -2,086.54 | 0.05 |
|  | Tide | 13 | 4,203.62 | 99.97 | 0.00 | -2,088.67 | 0.05 |
|  | Null | 2 | 4,390.95 | 287.29 | 0.00 | -2,193.47 | 0.00 |

^*^Three models did not converge during early migration period and were eliminated from the model set: (1) Blue Mussel + Coquina Clam + Crustacean + Miscellaneous Prey + Distance to Roost + TDF Count + Tide; (2) Crustacean + Miscellaneous Prey + Distance to Roost + Tide; (3) Blue Mussel + Coquina Clam + Crustacean + Miscellaneous Prey + Distance to Roost.

^a^ DF = Degrees of freedom.

^b^ AIC_c_ = Akaike’s Information Criterion corrected for sample size.

^c^ ΔAIC_c_ = Difference between a model’s AIC and that of the best fitting model.

^d^ *w_i_*^d^ = Akaike model weight.

^e^ LL = Log-Likelihood.

^f^ GOF = Goodness of fit = [log-likelihood(null model) – log-likelihood(model)]/log-likelihood(null model).

^g^ Miscellaneous Prey = Sum of horseshoe crab eggs (*Limulus polyphemus*), angel wing clams (*Cyrtopleura costata*), and other organisms (e.g., insect larvae, snails, worms).

^h^ All Prey = Sum of coquina clams + blue mussels + crustaceans + miscellaneous prey.

^i^ TDF Count = Tierra del Fuego Count = Counts of red knots using Tierra del Fuego wintering grounds by year (i.e., as an index for the total number of red knots in the flyway).
