# Supplementary material for: Factors that affect migratory Western Atlantic red knots (Calidris canutus rufa) and their prey during spring staging on Virginia’s barrier islands
Source: PLoS One. 2022 Jul 1;17(7):e0270224. doi: 10.1371/journal.pone.0270224 (PMC9249208; doi:10.1371/journal.pone.0270224)
Supplement: S5 Table — All models contained “Island” and “Year” as random effects. (DOCX) [file pone.0270224.s006.docx]

**S5 Table.** Full model sets for generalized linear mixed-effects regression models predicting crustacean, coquina clam, and miscellaneous prey abundances (organisms/m^2^) captured in 10 cm diameter x 3.5 cm deep cores on peat banks early in red knot migration (May 14 – 20, 2008 – 2018; *n =* 457; ‘early’) and on sand and peat banks at the approximate peak of red knot migration (May 21 – 27, 2007 – 2018; *n =* 1,322; ‘peak’), Virginia’s barrier islands. All models contained “Island” and “Year” as random effects.

| **Period** | **Prey** | **Model** | **DF^a^** | | **AIC_c_ ^b^** | | **ΔAIC_c_ ^c^** | ***w_i_*^d^** | | **LL^e^** | **MR2^f^** | **CR2^g^** |
| --- | --- | --- | --- | --- | --- | --- | --- | --- | --- | --- | --- | --- |
| Early | Crustacean | Tide + Water Temperature | 6 | | 110,491.00 | | 0.00 | | 0.61 | -55,239.42 | 0.02 | 0.57 |
|  |  | Tide + Water Temperature + Island Type | 7 | | 110,491.90 | | 0.88 | | 0.39 | -55,238.83 | 0.06 | 0.56 |
|  |  | Tide | 5 | | 113,026.10 | | 2,535.07 | | 0.00 | -56,507.99 | 0.02 | 0.58 |
|  |  | Tide + Island Type | 6 | | 113,027.30 | | 2,536.24 | | 0.00 | -56,507.55 | 0.05 | 0.57 |
|  |  | Water Temperature | 4 | | 119,599.50 | | 9,108.47 | | 0.00 | -59,795.71 | 0.00 | 0.57 |
|  |  | Water Temperature + Island Type | 5 | | 119,600.70 | | 9,109.62 | | 0.00 | -59,795.26 | 0.03 | 0.56 |
|  |  | Null | 3 | | 121,090.60 | | 10,599.54 | | 0.00 | -60,542.26 | 0.00 | 0.57 |
|  |  | Island Type | 4 | | 121,091.90 | | 10,600.87 | | 0.00 | -60,541.91 | 0.03 | 0.57 |
|  | Coquina Clam | Tide + Water Temperature | 6 | | 1,952.17 | | 0.00 | | 0.71 | -969.99 | 0.01 | 0.00 |
|  |  | Tide + Water Temperature + Island Type | 7 | | 1,954.23 | | 2.06 | | 0.25 | -969.99 | 0.01 | 0.01 |
|  |  | Tide | 5 | | 1,958.56 | | 6.39 | | 0.03 | -974.21 | 0.00 | 0.00 |
|  |  | Tide + Island Type | 6 | | 1,960.60 | | 8.43 | | 0.01 | -974.21 | 0.00 | 0.01 |
|  |  | Water Temperature | 4 | | 1,991.88 | | 39.71 | | 0.00 | -991.90 | 0.01 | 0.00 |
|  |  | Water Temperature + Island Type | 5 | | 1,993.91 | | 41.74 | | 0.00 | -991.89 | 0.01 | 0.01 |
|  |  | Null | 3 | | 1,997.67 | | 45.50 | | 0.00 | -995.81 | 0.00 | 0.01 |
|  |  | Island Type | 4 | | 1,999.70 | | 47.53 | | 0.00 | -995.81 | 0.00 | 0.00 |
|  | Misc. Prey^h^ | Tide + Water Temperature | 6 | | 8,721.57 | | 0.00 | | 0.71 | -4,354.69 | 0.04 | 0.69 |
|  |  | Tide + Water Temperature + Island Type | 7 | | 8,723.40 | | 1.84 | | 0.29 | -4,354.58 | 0.07 | 0.66 |
|  |  | Water Temperature | 4 | | 8,797.22 | | 75.65 | | 0.00 | -4,394.57 | 0.02 | 0.67 |
|  |  | Water Temperature + Island Type | 5 | | 8,799.07 | | 77.51 | | 0.00 | -4,394.47 | 0.04 | 0.68 |
|  |  | Tide | 5 | | 8,816.72 | | 95.15 | | 0.00 | -4,403.29 | 0.01 | 0.69 |
|  |  | Tide + Island Type | 6 | | 8,818.59 | | 97.03 | | 0.00 | -4,403.20 | 0.03 | 0.66 |
|  |  | Null | 3 | | 8,868.55 | | 146.99 | | 0.00 | -4,431.25 | 0.00 | 0.68 |
|  |  | Island Type | 4 | | 8,870.43 | | 148.87 | | 0.00 | -4,431.17 | 0.02 | 0.67 |
| Peak | Crustacean | Tide + Substrate + Water Temperature | 8 | 61,060.87 | | 0.00 | | | 0.56 | -30,522.38 | 0.02 | 0.22 |
|  |  | Tide + Substrate + Water Temperature + Island Type | 9 | 61,061.39 | | 0.51 | | | 0.44 | -30,521.62 | 0.05 | 0.23 |
|  |  | Tide + Substrate | 7 | 61,173.29 | | 112.41 | | | 0.00 | -30,579.60 | 0.01 | 0.24 |
|  |  | Tide + Substrate + Island Type | 8 | 61,173.48 | | 112.61 | | | 0.00 | -30,578.69 | 0.05 | 0.24 |
|  |  | Tide + Water Temperature | 7 | 62,545.87 | | 1,484.99 | | | 0.00 | -31,265.89 | 0.01 | 0.23 |
|  |  | Tide + Water Temperature + Island Type | 8 | 62,547.20 | | 1,486.32 | | | 0.00 | -31,265.55 | 0.05 | 0.23 |
|  |  | Tide | 6 | 62,705.37 | | 1,644.49 | | | 0.00 | -31,346.65 | 0.01 | 0.23 |
|  |  | Tide + Island Type | 7 | 62,706.46 | | 1,645.58 | | | 0.00 | -31,346.19 | 0.05 | 0.23 |
|  |  | Substrate + Water Temperature | 5 | 62,790.91 | | 1,730.03 | | | 0.00 | -31,390.43 | 0.00 | 0.22 |
|  |  | Substrate + Water Temperature + Island Type | 6 | 62,791.54 | | 1,730.67 | | | 0.00 | -31,389.74 | 0.04 | 0.22 |
|  |  | Substrate | 4 | 62,852.65 | | 1,791.78 | | | 0.00 | -31,422.31 | 0.00 | 0.22 |
|  |  | Substrate + Island Type | 5 | 62,853.09 | | 1792.21 | | | 0.00 | -31,421.52 | 0.04 | 0.22 |
|  |  | Water Temperature | 4 | 64,072.87 | | 3,011.99 | | | 0.00 | -32,032.42 | 0.00 | 0.23 |
|  |  | Water Temperature + Island Type | 5 | 64,074.20 | | 3,013.32 | | | 0.00 | -32,032.08 | 0.04 | 0.22 |
|  |  | Null | 3 | 64,171.97 | | 3,111.10 | | | 0.00 | -32,082.98 | 0.00 | 0.22 |
|  |  | Island Type | 4 | 64,173.14 | | 3,112.26 | | | 0.00 | -32,082.55 | 0.03 | 0.22 |
|  | Coquina Clam | Tide + Substrate + Water Temperature | 8 | 35,893.06 | | 0.00 | | | 0.69 | -17,938.47 | 0.04 | 0.26 |
|  |  | Tide + Substrate + Water Temperature + Island Type | 9 | 35,894.64 | | 1.58 | | | 0.31 | -17,938.25 | 0.05 | 0.26 |
|  |  | Tide + Water Temperature | 7 | 36,087.31 | | 194.25 | | | 0.00 | -18,036.61 | 0.02 | 0.27 |
|  |  | Tide + Water Temperature + Island Type | 8 | 36,088.72 | | 195.66 | | | 0.00 | -18,036.31 | 0.03 | 0.27 |
|  |  | Tide + Substrate | 7 | 36,271.95 | | 378.89 | | | 0.00 | -18,128.93 | 0.03 | 0.25 |
|  |  | Tide + Substrate + Island Type | 8 | 36,273.66 | | 380.60 | | | 0.00 | -18,128.78 | 0.03 | 0.25 |
|  |  | Tide | 6 | 36,496.63 | | 603.57 | | | 0.00 | -18,242.28 | 0.01 | 0.25 |
|  |  | Tide + Island Type | 7 | 36,498.21 | | 605.15 | | | 0.00 | -18,242.06 | 0.02 | 0.25 |
|  |  | Substrate + Water Temperature | 5 | 36,750.74 | | 857.69 | | | 0.00 | -18,370.35 | 0.03 | 0.25 |
|  |  | Substrate + Water Temperature + Island Type | 6 | 36,752.23 | | 859.17 | | | 0.00 | -18,370.08 | 0.03 | 0.25 |
|  |  | Water Temperature | 4 | 36,911.93 | | 1,018.88 | | | 0.00 | -18,451.95 | 0.01 | 0.25 |
|  |  | Water Temperature + Island Type | 5 | 36,913.27 | | 1,020.21 | | | 0.00 | -18,451.61 | 0.02 | 0.25 |
|  |  | Substrate | 4 | 37,159.65 | | 1,266.59 | | | 0.00 | -18,575.81 | 0.02 | 0.23 |
|  |  | Substrate + Island Type | 5 | 37,161.27 | | 1,268.22 | | | 0.00 | -18,575.61 | 0.02 | 0.23 |
|  |  | Null | 3 | 37,348.14 | | 1,455.08 | | | 0.00 | -18,671.06 | 0.00 | 0.23 |
|  |  | Island Type | 4 | 37,349.65 | | 1,456.59 | | | 0.00 | -18,670.81 | 0.01 | 0.23 |
|  | Blue Mussel | Tide + Substrate + Water Temperature + Island Type | 9 | 22,816.70 | | 0.00 | | | 0.51 | -11,399.28 | 0.06 | 0.07 |
|  |  | Tide + Substrate + Water Temperature | 8 | 22,816.77 | | 0.07 | | | 0.49 | -11,400.33 | . | . |
|  |  | Tide + Substrate | 7 | 22,898.73 | | 82.03 | | | 0.00 | -11,442.32 | . | . |
|  |  | Tide + Substrate + Island Type | 8 | 22,899.02 | | 82.32 | | | 0.00 | -11,441.46 | 0.03 | 0.03 |
|  |  | Tide + Water Temperature + Island Type | 8 | 26,026.80 | | 3,210.10 | | | 0.00 | -13,005.35 | 0.05 | 0.07 |
|  |  | Tide + Water Temperature | 7 | 26,027.23 | | 3,210.53 | | | 0.00 | -13,006.57 | 0.00 | 0.00 |
|  |  | Tide | 6 | 26,184.13 | | 3,367.43 | | | 0.00 | -13,086.04 | 0.00 | 0.00 |
|  |  | Tide + Island Type | 7 | 26,184.23 | | 3,367.53 | | | 0.00 | -13,085.07 | 0.00 | 0.00 |
|  |  | Substrate + Water Temperature + Island Type | 6 | 27,006.40 | | 4,189.70 | | | 0.00 | -13,497.17 | 0.04 | . |
|  |  | Substrate + Water Temperature | 5 | 27,006.97 | | 4,190.27 | | | 0.00 | -13,498.46 | 0.00 | 0.00 |
|  |  | Substrate + Island Type | 5 | 27,127.18 | | 4,310.48 | | | 0.00 | -13,558.57 | . | . |
|  |  | Substrate | 4 | 27,127.26 | | 4,310.56 | | | 0.00 | -13,559.62 | 0.03 | 0.03 |
|  |  | Water Temperature + Island Type | 5 | 33,593.99 | | 10,777.29 | | | 0.00 | -16,791.97 | . | . |
|  |  | Water Temperature | 4 | 33,595.12 | | 10,778.42 | | | 0.00 | -16,793.54 | 0.00 | 0.00 |
|  |  | Island Type | 4 | 33,774.91 | | 10,958.21 | | | 0.00 | -16,883.44 | 0.00 | 0.00 |
|  |  | Null | 2 | 46,762.83 | | 23,946.13 | | | 0.00 | -23,379.41 | 0.00 | 0.00 |

^a^ DF = Degrees of freedom.

^b^ AIC_c_ = Akaike’s Information Criterion corrected for sample size.

^c^ ΔAIC_c_ = Difference between a model’s AIC and that of the best fitting model.

^d^ *w_i_*^d^ = Akaike model weight.

^e^ LL = Log-Likelihood.

^f^ MR2 = Marginal r-squared = Considers only the variance of the fixed effects.

^g^ CR2 = Conditional r-squared = Considers the variance of both fixed and random effects.

^h^ Misc. Prey = Miscellaneous Prey = Sum of horseshoe crab eggs (*Limulus polyphemus*), angel wing clams (*Cyrtopleura costata*), and other organisms (e.g., insect larvae, snails, worms).
